# Immobilization strategies for porphyrin-based molecular catalysts for the electroreduction of CO_2_

**DOI:** 10.1039/d2ta00876a

**Published:** 2022-03-17

**Authors:** Maryam Abdinejad, Keith Tang, Caitlin Dao, Saeed Saedy, Tom Burdyny

**Affiliations:** a Department of Chemical Engineering, Delft University of Technology Van der Maasweg 9 2629 HZ Delft The Netherlands M.Abdinejad@tudelft.nl T.E.Burdyny@TUDelft.nl; b Department of Physical and Environmental Sciences, University of Toronto Scarborough 1265 Military Trail Toronto ON M1C 1A4 Canada

## Abstract

The ever-growing level of carbon dioxide (CO_2_) in our atmosphere, is at once a threat and an opportunity. The development of sustainable and cost-effective pathways to convert CO_2_ to value-added chemicals is central to reducing its atmospheric presence. Electrochemical CO_2_ reduction reactions (CO_2_RRs) driven by renewable electricity are among the most promising techniques to utilize this abundant resource; however, in order to reach a system viable for industrial implementation, continued improvements to the design of electrocatalysts is essential to improve the economic prospects of the technology. This review summarizes recent developments in heterogeneous porphyrin-based electrocatalysts for CO_2_ capture and conversion. We specifically discuss the various chemical modifications necessary for different immobilization strategies, and how these choices influence catalytic properties. Although a variety of molecular catalysts have been proposed for CO_2_RRs, the stability and tunability of porphyrin-based catalysts make their use particularly promising in this field. We discuss the current challenges facing CO_2_RRs using these catalysts and our own solutions that have been pursued to address these hurdles.

## Abstract 2

Using homogeneous and heterogeneous metalloporphyrin-based catalysts for electroreduction of CO_2_ to value-added materials.

## Graphical abstract

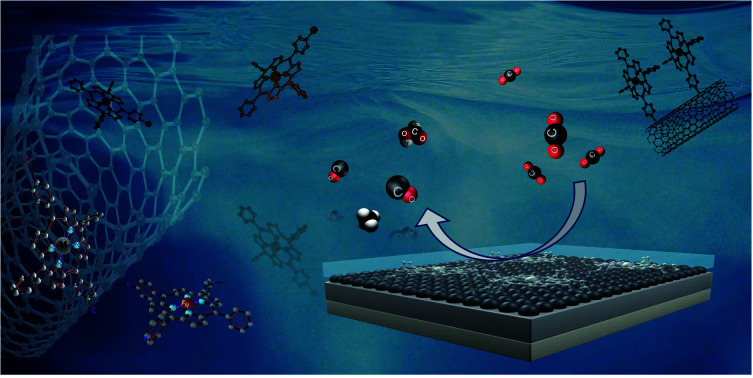

## Introduction

Carbon dioxide (CO_2_) conversion technology is emerging as a promising tool to aid in the quest to lower CO_2_ emissions.^1,2^ Current advances have successfully converted CO_2_ to small C1 building block chemicals: CO,^3^ CH_4_,^4^ formaldehyde^5^ and formic acid;^6–8^ high energy dense liquid fuels: methanol (MeOH),^9^ ethylene (CH_2_CH_2_),^10^ ethanol,^11^ petrochemical polymers,^12^ and hydrogels.^13^ The abundance and cost efficiency of CO_2_ as a resource, makes its conversion economically viable as a competitor to traditional methods of manufacturing (*e.g.*, carbonylation^14^ and the methanol to olefin (MTO) process^15–17^). Research efforts to further utilize captured CO_2_ as a raw material for the production of higher value compounds and chemical feedstocks have intensified in recent years with the advent of efficient electrolyzer technologies and heterogenization techniques. Electrochemical (EC) and photoelectrochemical (PEC) CO_2_ reduction technologies are the leading approaches to achieve CO_2_ reduction.^18,19^

Electrocatalysts are instrumental to CO_2_RR due to their contributions to overcoming kinetic energy barriers and in mediating Proton Coupled Electron Transfers (PCETs).^20–22^ Benchmarks for potential commercial implementation stipulate the necessity of high current densities (*j* > 200 mA cm^−2^), long operation capacities, high selectivities (>90%), and low overpotentials.^23^ Although the catalytic capabilities of proposed systems have improved considerably, their current state remains insufficient for industrial/commercial application. Compared to the initial performance of catalysts, their long-term stability needs to be considered. Based on the techno-economic analysis, the stability of electrocatalysts for CO_2_RR should be at least 4000 hours.^24,25^ The stability of the catalytic system depends on multiple factors including catalyst's structure, catalyst immobilization including chemical and physical including covalent and non-covalent bonding, support material, catalyst's loading, catalyst surface morphology and the type of metal centre in the case of metallo-porphyrins.^26,27^

Advances in electrochemical CO_2_ reduction reactions (CO_2_RRs) offer a realistic pathway to utilization of CO_2_ as an abundant and inexpensive source for C1 building blocks.^28^ Until recently, solid state electrocatalysts had been leading the field in terms of conversion efficiency (current density). Often composed of heavy metals such as Pt, Pd, Au, Ag, Cu, *etc.*, yet costly to implement and maintain.^29–32^ Although solid state catalysts have proven themselves capable of reducing CO_2_ to energy dense compounds like MeOH, ethylene, and ethanol; there is much to be desired for their selectivity for the reduction products they produce.^33^

On the other hand, molecular catalysts^34,35^ are favoured for their high selectivity and are capable of converting CO_2_ to CO,^3^ formaldehyde,^36^ formic acid,^37^ oxalic acid/oxalate,^38^ cyclic carbonates,^39^*etc.* with selectivities at near unity. Macrocyclic tetrapyrrolic ligands such as porphyrins and phthalocyanines are used as molecular catalysts,^40^ and often incorporate earth-abundant metals such as Fe,^41^ Cu,^42,43^ Co,^44–46^ Ni,^47^ Zn^48^*etc.*, which are popular due to their high stability and facile tunability.^49–53^ Additionally, the highly conjugated system of porphyrin-based molecules result in a number of invaluable properties, such as enhanced electronic conductivity and π–π stacking capabilities.^54–57^

Although there exist several overarching reviews of CO_2_ electrocatalysts including even more specific reviews on porphyrin/phthalocyanine catalysts,^50,51^ a coverage of more recent developments in the field is required. Herein, major advances involving the functionalization of porphyrin and phthalocyanine catalysts for electrochemical CO_2_RR will be recounted in detail with a focus on recent advances in heterogeneous electrocatalysts and the effects of various immobilization strategies on catalytic performance.

## Mechanistic pathways of CO_2_RR

The identity of the metal centre plays a significant role in the activity and selectivity of the catalyst. In the first step of the CO_2_RR mechanism, the electrophilic C atom is activated by nucleophilic attack from an electron-rich metal centre. The initial binding of CO_2_ requires the C–O σ* (LUMO) and degenerate C–O π* (LUMO+1) orbitals on the C atom be filled with electrons from the metal center.^57,58^ To satisfy this condition, an M^+1,0^ centre with a d^8^ configuration in a square-pyramidal ligand field is best for binding CO_2_*via* its filled d_*z*_^2^ (σ) and d_*xz*/*yz*_ (π back-bonding) orbitals. For this reason, Fe, Co and Ni are hypothesized to be the best metals for catalysis due to their d^8^ electron configuration. Product selectivity is then influenced by the ability of CO to remain adsorbed to the metal for further reduction or desorption, leading to the release of CO.^59^ It has been proposed that Fe, Co, and Ni contain doubly occupied d_*z*_^2^ orbitals that would repel the lone pair of electrons on CO after CO_2_ reduction, releasing CO as the major product.^60^ However, if the CO remains bound to the metal *via* σ bonding, further reduction can take place, producing CH_4_. In metals having outermost s or p electrons, the electron transfer happens at the more localized, lower energy orbital, which is not strong enough to reduce CO, leading to the production of [CO_2_] followed by further reduction to formic acid depending on the availability of the proton source.

The prediction from the molecular orbital theory closely resembles to the qualitative results observed in the literature. For example, the effect of the metal centre on CO_2_RR has been studied in extensively with 17 different metallophthalocyanines (MPcs) using gas diffusion electrodes (GDEs).^61^ Co, Ni, Fe, and Pd, belonging to group VIII of the transition elements, generate CO as the main CO_2_RR product. Co and Ni in particular, were most impressive with current efficiencies of 98 and 100% respectively (between −1.0 and −1.75 V (*vs.* RHE)). Sn, Pb, In, Zn, and Al produce formic acid as the main product, with Zn also being able to generate CO to a comparable extent. Cu, Ga, and Ti are unique in being the only metals that give methane as the main product, with current efficiencies being as large as 30–40%, while methane production for other metals is almost negligible. Lastly, V, Mn, Mg, Pt, and H show poor activity for CO_2_RR, with competing hydrogen evolution at current efficiencies of 90–100%. Although the selectivity of these metals towards one product is favourable, the activity must also be considered. CoPc and FePc showed higher current densities at lower applied potentials, while NiPc, although having a high selectivity for CO, requires much more energy to drive similar CO current densities. The high activity and selectivity of Co porphyrins towards CO makes the use of Co porphyrins one of the most promising catalysts for CO_2_RR, and therefore, has been subject to many detailed investigations.^62,63^ Although, closely following behind are NiPc and FePc, which are also promising catalysts if activity could be increased in the case of NiPc, and selectivity is enhanced in the case of FePc.^64^

**Fig. 1:**
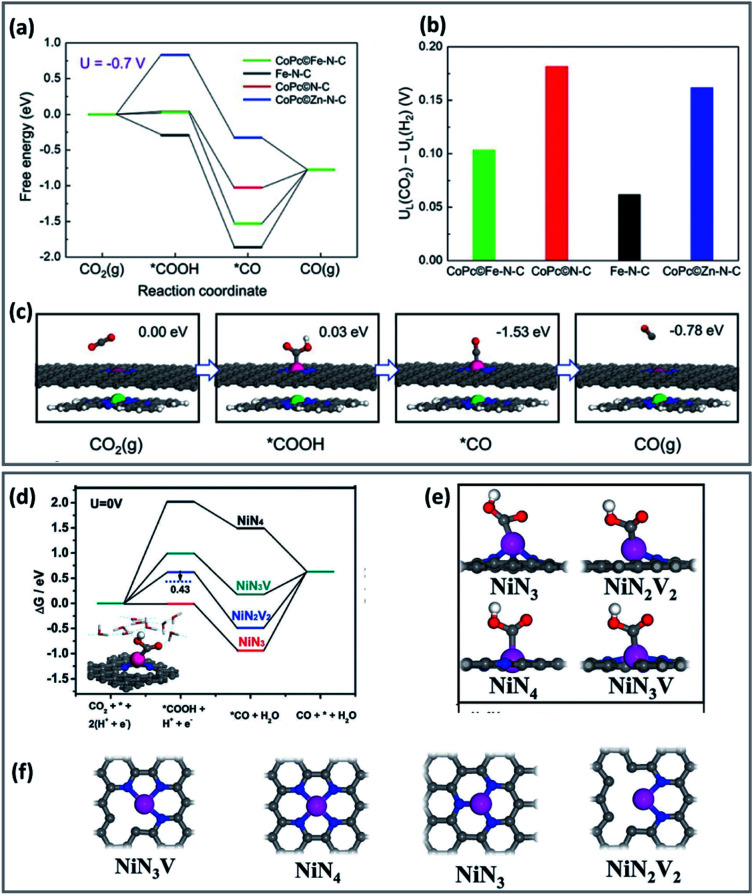
Computational results of CO_2_RR process on different catalysts. (a) Free energy diagram for the CO_2_RR to CO at *U* = −0.7 V *vs.* RHE on the Fe site in CoP/Fe–N–C, Fe site in Fe–N–C, Co site in CoP/N–C, and Zn site in CoP/Zn–N–C, respectively. (b) The differences in limiting potentials for CO_2_RR (*U*_L_ (CO_2_)) and HER (*U*_L_ (H_2_)) on the active sites. (c) Schematic atomic structure of CO_2_RR process on the Fe site in CoPc/Fe–N–C with the free energies at *U* = −0.7 V *versus* RHE. DFT calculations (copyright 2019 Wiley-VCH).^65^ (d) Free energy diagrams solvation effect corrections for the CO_2_RR and the HER. (e and f) Optimized atomic structures of different Ni–N structures with Ni atoms coordinated with 4 N atoms (NiN_4_), 3 N atoms (NiN_3_ and NiN_3_V), 2 N atoms (NiN_2_V_2_) (copyright 2012 Science).^67^

Incorporation of a metal active canter to porphyrin/phthalocyanine-based catalysts promotes a cooperative effect between the catalyst metal site and the metal electrode. For instance, combining CoPc and Fe single-atom sites, showed that the free energy decreased in the activation and desorption steps of CO_2_RR (Fig. 1a–c).^65^ In this case, CoPc molecules reduced the adsorption energy of *CO and H*, without weakening the formation activity of *COOH. Therefore, combining CoPc and Fe–NC enhances the CO_2_RR activity on the Co centre while reducing the adsorption of CO on the Fe site.^66^

In another study, theoretical calculations indicate that a lower coordination number could change the electronic structure of the active site and increase the success of CO_2_RR over HER (Fig. 1d–f).^67^ The free energy of *COOH of coordinated, unsaturated NiN_3_, NiN_3_V, and NiN_2_V_2_ is lower than that of saturated NiN_4_. The *H blocking was also relatively weak in the case of NiN_3_V and NiN_2_V_2_, and for NiN_2_V_2_, resulting in high product selectivity.

The effect of the ligand can drastically affect the activity of these catalysts. Strategies for improving homogeneous catalysts include introducing functional groups that serve as local proton sources,^68,69^ hydrogen bond donors,^70^ or cationic moieties in the second sphere environment,^71,72^ all of which have resulted in increased CO_2_RR rates in part due to the stabilization of the formed CO_2_ intermediate by the substituents on the porphyrin ligand. A classic strategy of improving performance was pioneered by Savéant's group on Fe porphyrins through structural modification of the porphyrin ligand by incorporating substituents that can induce through-structure electronic effects.^73^ Introducing electron-withdrawing groups such as fluorine atoms has been shown to decrease overpotential by lowering electron density near the metal active site, making it is easier to inject an electron into the catalyst. However, this may also subsequently decrease catalytic activity by decreasing the nucleophilicity of the metal and its ability to bind to CO_2_. On the other hand, the introduction of methoxy substituents increases catalytic activity by increasing the propensity of the metal center to bind to CO_2_*via* inductive electron-donating effects of the ligand. Careful balance of electron-donating (lowering overpotential) and electron-withdrawing (increasing TOF) is required in ligand design and an optimal push–pull system is needed to achieve the ideal molecular catalyst.^74^

In the case of heterogeneous catalysts however, the effect of electron withdrawing (*i.e.* F and CN)^75,76^ and electron-donating (*i.e.* octaalkoxyl)^77^ substituents show little improvement for CO_2_RR in terms of the desired electronic effect. However, these substituents are crucial in reducing aggregation and reducing π–π stacking interactions that lead to improved catalytic activity. Ligand modification within the context of heterogenized molecular catalysts presumably affects other factors in the immobilized catalyst system including electron transfer between the catalyst and electrode, the ability for CO_2_ to coordinate with the catalyst, the desorption rate of the reduced products, and solvation energies.^26^ These findings emphasize the need to not screen heterogeneous molecular catalysts by the same criteria as homogeneous catalysts alone.

## Homogeneous *vs.* heterogeneous electrocatalysts

Molecular catalysts can be applied in two general categories: as either homogeneous or heterogeneous systems.^78^ Whereas heterogeneous catalysts exist in a separate physical phase from the reactant (CO_2_), homogeneous systems operate in the same phase as the reactant. Homogeneous studies are a convenient way to assess the initial CO_2_ reduction ability of novel molecular catalysts. Oftentimes, only those that show promise under these conditions are further investigated with more vigorous heterogeneous studies. Hu *et al.*^79^ make a compelling argument for a reassessment of this method of screening, that can sometimes allow promising but underperforming molecules to slip through the cracks. Their report of cobalt tetraphenylporphyrin (CoTPP) immobilized onto carbon nanotubes (CNTs) illustrates how CoTPP, a catalyst whose activity is traditionally eclipsed by iron tetraphenylporphyrin (FeTPP) in homogeneous conditions, performs significantly better when immobilized onto a conductive CNT support in aqueous media (FEco = 83%; *j* = −0.59 mA cm^−2^ at −1.15 V *vs.* SCE) than an analogous FeTPP–CNT (FEco = 64%; *j* = −0.9 mA cm^−2^).^80^ They propose a new, simple deposition method consisting of sonicating the dissolved catalysts and CNTs, drop casting the solution, and drying as a means to quickly screen new molecular catalysts.

Comprehensive studies comparing identical catalysts in homogeneous and heterogeneous environments widely demonstrate an overall enhancement to catalytic performance upon immobilization onto electron conductive supports.^76,79^ Systematic studies show that a significant enhancement in catalytic reactivity was achieved through immobilization of Fe-TPP-dimers onto CNTs in aqueous solution (TOF = 10 s^−1^; FE_CO_ = ∼90%) compared to their homogeneous analogues in DMF (TOF = 0.11 s^−1^; FE_CO_ = 48% at −1.33 V *vs.* RHE).^81^ We have also shown that heterogeneous pyridine–porphyrin complexes exhibit higher catalytic activity and product selectivity (FE_total_ > 92% and *j* = −30 mA cm^−2^ at −0.6 V *vs.* RHE) compared to their homogeneous counterparts (FE_total_ = 76% and *j* = −1.34 mA cm^−2^ at −1.4 V *vs.* RHE).^74^

Heterogeneous immobilization of molecular catalysts onto conductive solid supports is advantageous in several ways: (1) unlike in homogeneous systems, immobilized catalysts are locally bound to the electrode, the source of reductive capability, guaranteeing a high degree of catalytic site exposure.^82,83^ This serves to streamline the pathway of electron transfer from the electrode to the catalytically active site to CO_2_; (2) moreover, the solid support is often chosen by virtue of its exceptional electrical conductivity, further ensuring efficient electron transfer processes;^84,85^ (3) most organic/inorganic molecules are limited by their solubility in aqueous solvents. Heterogeneous systems enable molecular catalysts to overcome such limitations, freeing them to operate in proton-rich aqueous solutions, which serve a dual purpose in being more green.^69,86^ These strategies have proven to be a promising approach to efficiently enhancing catalytic activity. In the previous study of pyridine–porphyrin complexes, we demonstrate that even with a lower catalyst load concentration, the performance of heterogeneous molecules on CNTs is superior to that of its homogeneous analog.^74^

Converting CO_2_ into value-added materials is thought to occur *via* several mechanistic pathways. After capturing CO_2_, an initial proton coupled electron transfer (PCET) process forms intermediates such as *COOH and *OCHO. Among various carbonaceous products, CO and formic acid are considered pivotal C1 building blocks for C_2+_ products. The formation of C_2+_ products is much more challenging due to the number of reaction steps and intermediates required to form the C–C bond. This difficulty is also due to the linear relationship between the binding energies of individual reaction intermediates and their activation energies (kinetic barrier).^87,88^ Given the competition of C–C coupling with H–H and C–H bond formation,^89^ strategies that improve CO* dimerization to OC–CO* is key to the production of C_2+_ products. General strategies to achieve this include manipulating CO* binding strength through catalytic design, increasing CO* coverage, controlling CO* adsorption energetics,^87^ and re-adsorbing electrogenerated CO.^90^

To achieve C–C bond formation, the adsorbed *CO species may interact with each other *via* the Langmuir–Hinshelwood (LH) step through surface-bound species and a species in solution described in the Eley–Rideal (ER) step.^91^ In the case of metallo-porphyrins, the metal active site needs to bind to the *CO intermediates strongly enough to facilitate C–C coupling, but not too much as to significantly increase the energy barriers. Fundamental theoretical studies are valuable when designing catalysts.^92^ Li *et al.*^93^ demonstrated the potential of molecule-enhanced surfaces and how the CO_2_ to CO conversion efficiency of 5,10,15,20-tetraphenyl-21*H*,23*H*-porphine iron(iii) chloride (FeTPP[Cl]) contributes to enhanced C_2_ production on a Cu electrode. They were able to utilize immobilized FeTPP[Cl] to create a localized concentration of ·CO, which serves as a key intermediate for the Cu active sites in the production of ethanol. By showing that the binding energy of CO to FeTPP[Cl] was 0.2 eV weaker than that of the Cu (111) substrate, the authors hypothesized that the CO produced by FeTPP[Cl] was readily spilling over onto the Cu active sites.

## Noncovalent electrode immobilization by adsorption

Non-covalent immobilization relies on the π–π interactions from the conjugated aromatic system that exists on aromatic macrocycles to bind to carbon surfaces.^76,94,95^ Porphyrins and phthalocyanines being aromatic macrocycles are good candidates for surface immobilization due to their strong π–π interactions (Table 1). These interactions lead to improved electron transport rates due to the closer proximity of the catalyst to the electrode and the potential for improved electron conductivity from the in-plane π–π stacking. Coverage of the electrode surface with molecular catalysts may minimize its contact with water and reduce the opportunity for HERs.^96^ This method has been used for different applications such as water oxidation^97^ and proton reduction^98^ in addition to electrocatalytic reduction of CO_2_ to CO.^76,79,99^

**Table 1:** Summary of non-covalently immobilized heterogeneous molecular electrocatalysts for electrocatalytic CO_2_ reduction

Catalyst	*V vs.* RHE	*j* (mA cm^−2^)	FE% (CO)	TOF (s^−1^)	Ref.
Fe-TPP	−0.8	7.8	54%	0.02	86
Fe-TPP–NH_2_	−0.8	12.9	79%	0.05	86
Fe-TPP–adj(NH_2_)_2_	−0.8	8.0	70%	0.03	86
Fe-Tetra-Py	−0.7	19.6	37	0.9	74
Fe-Cis-Py	−0.7	30.4	67	3.49	74
Fe-Tri-OMe-Py	−0.7	−23	50	1.49	74
Fe-TPP-dimer	−0.8	16	89	10.2	81
CoPc–CNT	−0.63	∼15	98	4.1	76
CoP–pc	−0.61	18	90	1.4	100
COF-367-Co	−0.67	3.3	90	0.53	50
CoPc–P4VP	−0.73	2.0	89	4.8	101
CoPc2	−0.67	18.1	93	6.8	95
Co-TPP	−0.8	0.9	70	2.75	79
Fe-CATpyr	−0.59	0.24	93	0.04	102
CAT_CO_2_H_	−1.35	0.4	80	0.05	103
CoFPC	−0.9	6.0	88	2.05	75
CoTAP	−0.8	2.5	86	2	104
Co-TPP	−0.9	0.8	52	4.5	105
FePGH	−0.4	2.8	96	0.8	106
NiPor-CTF	−0.9	42	97	0.47	107
N–CoMe2Pc/NRGO	−0.8	8.7	90	1	108
FePc-Gr75	−0.6	1.7	90	—	109
CoPc/Znln2S4	−0.83	8.1	92.6	—	110
PCN-222(Fe)/C	−0.6	1.2	91	1.2	111
Ni-TPP–NItBu	−0.5	22	94	7.2	47
Co-qpyCOOH/CNT	−0.65	6.7	100	0.28	112
CoTPyPP/CNT	−0.6	7.5	95	2.1	54

**Fig. 2:**
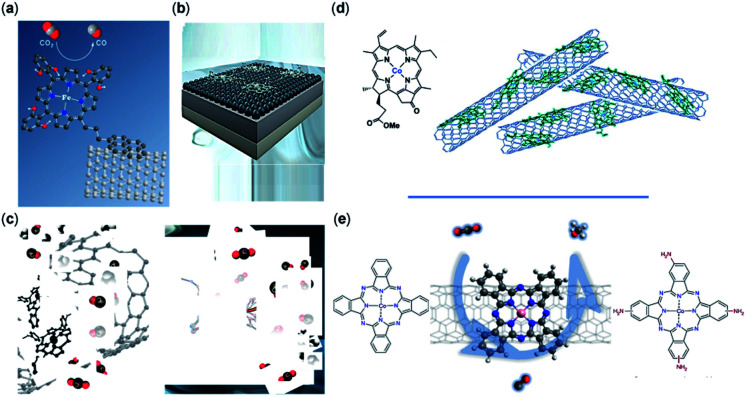
Non-covalent immobilization of porphyrin/phthalocyanine CO_2_RR catalysts onto carbon-based electrodes representative of the catalysts reported in (a) an iron triphenylporphyrin bearing 6 pendant –OH groups on the *ortho* positions of the phenyl rings immobilized on CNTs with a pyrene group (copyright 2016, Royal Society of Chemistry);^102^ (b) iron tetraphenylporphyrin immobilized in a flow cell (copyright 2020 Elsevier);^86^ (c) iron porphyrin–pyridine derivatives immobilized on CNTs (copyright 2020 American Chemical Society);^74^ (d) cobalt chlorin adsorbed on MWCNTs on a glassy carbon electrode (copyright Royal Society of Chemistry);^116^ and (e) cobalt phthalocyanine immobilized on CNTs (copyright 2019, Nature Publishing Group).^117^

The support material, surface functionality, morphology, and conductivity of the electrode are necessary for CO_2_RR and have been shown to enhance the catalytic efficiency, catalyst regeneration, and product separation.^113^ The support material and its interaction with the molecular catalyst directly affect the electron transfer, transport of species, the strength of catalyst bonding to the surface, and durability of catalyst; it also may alter the CO_2_RR mechanism.^114^ Highly conductive support ensures suitable electron transfer and reduces the ohmic resistance of the electrode, making high current densities possible.^76,79^ Carbon-based materials such as CNTs, carbon black (CB), carbon paper (CP), graphene derivatives, *etc.* are of particular interest for CO_2_RR due to their high stability and conductive surface area (Fig. 2).^76,79,94,97,98,102,115–117^ In another study, it has been reported that the CoPc catalysts immobilized on CNTs reveal an exceptional CO activity compared to CoPc immobilized onto other carbon-based materials such as reduced graphene oxide, carbon fiber paper, and CB.^76^

Several techniques may be used to achieve noncovalent hybridization, such as dip coating and drop-casting. These methods involve dissolving the catalyst and immersing the carbon-based support material in a suitable solvent such as DMF, followed by deposition of the mixture onto the desired surface. Suspension methods ensure a homogeneous dispersion of the catalyst throughout the solid support and minimize the chance of unfavourable molecular aggregation, which can inhibit electron delivery. Shen *et al.*^3^ propose a detailed mechanistic scheme for CO_2_ electroreduction to CO and CH_4_ with CoTPP immobilized onto pyrolytic graphite (PG). Their work emphasizes the importance of pH in facilitating the initial electron transfer that activates CO_2_, by demonstrating the pH dependency of CH_4_ production, as well as in minimizing H_2_ evolution, which is predominantly produced at low pH (pH = 1). They also identify the CO_2_ radical anion (CO_2_˙^−^) as the key reaction intermediate in CO production. Although the formation of CO_2_˙^−^ typically occurs at very negative potentials, a key strategy in successful catalytic systems lies in stabilizing its coordination to the catalyst. Using a narrow pH range (pH 1–3), they identified conflicting reaction pathways for the reaction products, where CO production is catalysed at pH = 3, and CH_4_ production is catalysed at pH = 1. They achieved 60% FE_CO_ at pH = 3, pressure = 10 atm, at −0.6 V *vs.* RHE and traces of CH_4_ (∼2.4% FE_CH_4__) at higher overpotentials (−0.8 V *vs.* RHE).

**Fig. 3:**
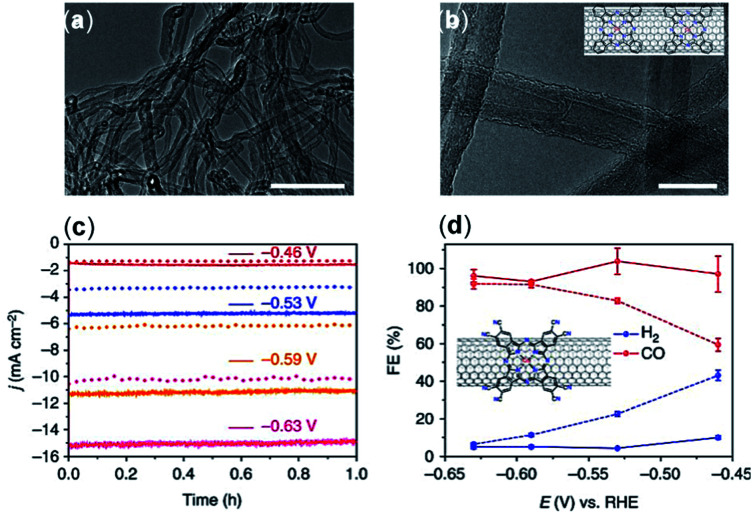
(a and b) TEM images of the CoPc/CNT (6%) hybrid. Inset in (b) shows a schematic representation of the CoPc/CNT hybrid. (c) Representative chronoamperograms of CO_2_ electroreduction catalyzed by the CoPc/CNT (2.5%) hybrid for 1 h at various potentials in 0.1 M KHCO_3_ aqueous solution; (d) FE of reduction products at different potentials for CoPc–CN/CNT (solid line) and CoPc/CNT (dotted line).^76^ Copyright 2017, Nature Publishing Group.

The surface morphology and graphitic degree of different materials should be considered when choosing a solid support. Wang *et al.*^76^ compared the catalytic activity of CoPc catalysts immobilized directly onto several carbon materials including CNTs, carbon fiber paper, reduced graphene oxide, and CB. Compared to CNTs, these other materials were found to have less than a third of the current density, ∼10% lower FEs, and inferior catalytic stability. The morphology of the immobilized CoPc/CNT can be visualized with transmission electron microscopy (TEM) (Fig. 3a and b). Aoi *et al.*^116^ found that a significant decline in FE_CO_ selectivity of a cobalt-porphyrin chlorin complex occurred when a graphene oxide matrix was used compared to when the same catalysts were deposited onto multi-wall CNTs (MWCNTs) in similar conditions. This decline in selectivity was attributed to the higher graphitic degree of CNTs, which resulted in increased π–π interactions between the molecular catalyst and the carbon support.^79^ In their study, Hu *et al.* noted a higher level of catalyst detachment occurring with a CB scaffold during electrolysis, whereas a comparable CNT support was more stable.

**Fig. 4:**
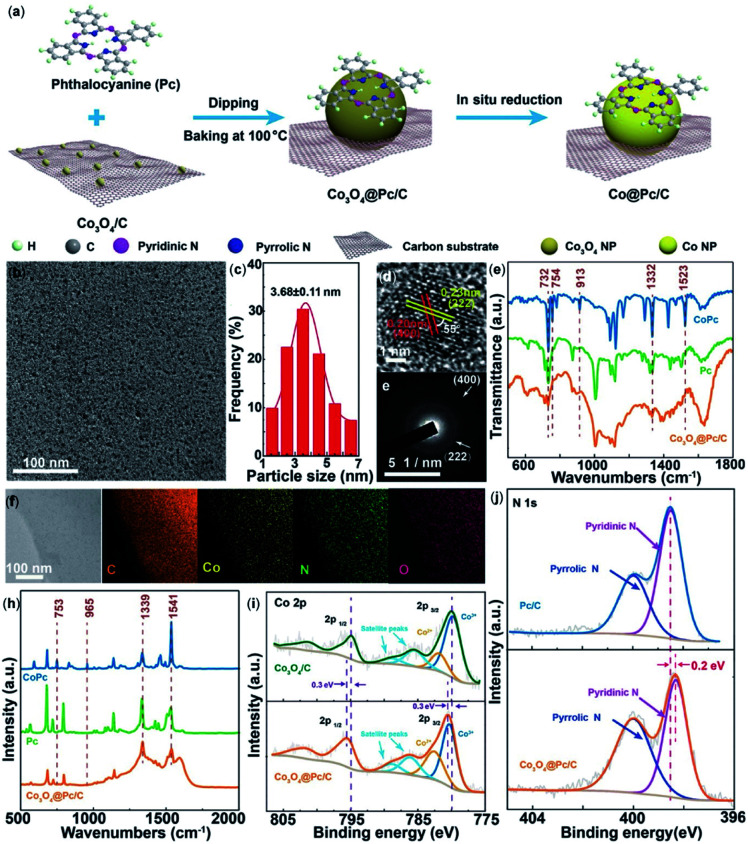
(a) An illustration of the synthetic procedure for Co@Pc/C; (b) TEM image; (c) size distribution histogram; (d) high-magnification TEM image; and the corresponding (e) fast Fourier-transform (FFT) pattern; (f) STEM-EDS elemental mapping images; and (h) Raman spectra of Co_3_O_4_@Pc/C alongside commercial CoPc and Pc; (i) high-resolution Co 2p XPS spectra of Co_3_O_4_@Pc/C and Co_3_O_4_/C. (j) High-resolution N 1s XPS spectra of Co_3_O_4_@Pc/C and Pc/C.^118^ Copyright 2020 Wiley-VCH.

In another recent report, an enhancement in electrochemical CO_2_RR of free base phthalocyanines was reported using N-doped carbon materials (N-Cmat).^118^ It was demonstrated that reduction of CO_2_ to CO occurred with the pyridinic N's as opposed to the pyrrolic N's. Introduction of Co nanoparticles, Co@Pc/C, led to CO production with a FE_CO_ of 84% and current density of 28 mA cm^−2^ at −0.9 V (Fig. 4).

**Fig. 5:**
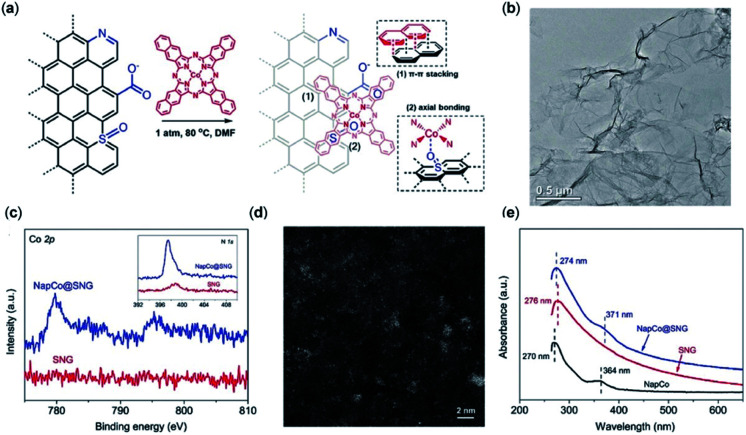
(a) Heterogenization of NapCo onto graphene through π–π stacking and heteroatom coordination; (b) bright-field TEM image of NapCo@S/N/O heteroatoms doped graphene (SNG); (c) XPS surveys of Co 2p core electron levels of SNG and NapCo@SNG. Inset: the N 1s core electron levels of SNG and NapCo@SNG; (d) HAADF-STEM images of NapCo@SNG where the Co atoms are indicated by the bright spots; (e) UV/Vis spectra of NapCo before and after immobilizing onto SNG.^119^ Copyright 2019 Wiley-VCH.

Other studies of immobilized Co^II^-2,3-naphthalocyanine (NapCo) complexes onto doped graphene in aqueous solution find that the electronic transfer processes between the catalyst and the conductive surface are improved through axial Co–O coordination to the terminal sulfoxide groups, resulting in a 3-fold increase to the TOF and a FE_CO_ of up to 97% (Fig. 5).^119^

Deposition of porphyrin molecules onto hydrophobic substrates such as polytetrafluoroethylene (PTFE) and Nafion has also seen success.^120,121^ The hydrophobic microenvironment of the polymer significantly enhances CO_2_ gas diffusion and mass transport, increasing the local concentration of CO_2_ on the electrode for CO_2_RR.^122^ Nafion is another example of a tetrafluoroethylene based polymer that possesses additional ionic properties due to its sulfonic acid groups which facilitates proton transfer for CO_2_ reduction. It was shown to work synergistically with carbon-based materials such as CNTs, demonstrating a ∼10 fold current enhancement for the reduction of CO_2_ to CO at −1.4 V *vs.* Ag/AgCl (pH 7).^123^ However, CO_2_ permeability through the Nafion membrane remains limited, resulting in lower FE and current density when used for CO_2_ reduction to formate.^124^

Early studies of covalent modification of an electrode surface with metalloporphyrins was reported by Aramata *et al.*^125^ in which Co-5,10,15,20-tetrakis(4-carboxylphenyl)porphyrin (CoTCPP) was fixed to a glassy carbon electrode functionalized with 4-aminopyridine groups *via* coordination of the Co centre with pyridine. The modified electrode demonstrated a FE_CO_ of 50% at −1.2 V *vs.* SCE in a CO_2_-saturated standard phosphate buffer solution (pH 6.8). Even after prolonged potentiostatic electrolysis under above conditions, the electrode remained stable, with no decrease in current density for more than 4 h. The authors attribute this improvement in catalytic activity to the increased electron density on the central Co(ii) ion after axial coordination to the electron-donating pyridine moiety, thereby stabilizing the binding of CO_2_ on the opposite coordination site.

A later study utilizes a similar strategy to immobilize Co phthalocyanine (CoPc) onto polymeric films composed of pyridines (poly-4-vinylpyridine or P4VP) *via* a coordination bond.^126^ The CoPc–P4VP films display a FE_CO_ of ∼90%, with a TOF of 4.8 s^−1^ at −0.75 *vs.* RHE, which is drastically improved over the CoPc alone, adsorbed onto an edge-plane graphite (EPG) electrode. The latter only displays a 36% FE_CO_ along with a TOF of 0.6 s^−1^. In addition to the increase in d_*z*_^2^ orbital energy from the axial coordination, the authors hypothesize the improvement in catalytic activity to be from the encapsulation of the porphyrin catalyst inside the polymer film. This leads to higher CO_2_ solubility in the otherwise hydrophobic membrane due to basic pyridine sites and the second sphere hydrogen bond/proton network provided by the ionizable pyridine groups.

## Covalent modification of electrode

Covalent immobilization establishes a direct bond between the molecular catalyst and the electrode surface (Table 2). This is beneficial in a number of ways. For one, the bond connecting the electrode to the catalyst layer can lead to heightened electron conductivity, and by extension more efficient use of energy (lower potentials).^127^ Secondly, covalent immobilization is a more robust alternative to non-covalent approaches which can show signs of catalyst displacement after several hours of operation.^108,115,128^ Here, the ligand groups of the porphyrin must be functionalized in a way that both allows for covalent binding to a surface, without destabilizing the molecule, while also remaining active for CO_2_RR.^112,129^ Such an approach provides the opportunity for long-term stability and predictable catalyst orientations, but leads to a high degree of constraints, generally adding complexity to the synthetic approach required.

**Table 2:** Summary of covalently immobilized heterogeneous molecular electrocatalysts for electrocatalytic CO_2_ reduction

Catalyst	*V vs.* RHE	*j* (mA cm^−2^)	FE% (CO)	TOF (s^−1^)	Ref.
CoTAP	−0.9	4	100	6	104
CAT_CO_2_H_	−0.8	0.6	86	0.05	130
CoPPCl	−0.8	25	98	1.9	131
Co-TPP	−0.8	1.5	67	8.3	105
COF-366-Co–CNT	−0.68	6.8	92	1.2	132
MWCNT-Por-COF-Co	−1.0	18.7	99.3	70.6	129

Covalent attachment of an electrocatalyst to a solid support has been shown to improve catalytic performance as demonstrated by Y.-F. Han *et al.*^131^ in which protoporphyrin IX cobalt chloride (CoPPCl) was covalently linked to hydroxyl-functionalized carbon nanotubes (CNT–OH). The grafted catalyst was synthesized by refluxing CoPPCl with CNT–OH (3.06 wt% hydroxyls) in ethanol with triethylamine, generating a covalent bond between the hydroxyl O atom and the Co center, and resulting in the functionalized material CoPP@CNT. The CoPP@CNT composite and Nafion were suspended in ethanol and drop cast onto carbon paper reaching a catalyst loading of 60 μg cm^−2^. The catalytic performance was then evaluated in a low-volume two-compartment cell with a CO_2_-saturated 0.5 M NaHCO_3_ electrolyte. The FE_CO_ of the CoPP@CNT composite ranges from 90% at −0.65 V to 80% at −0.5 V *vs.* RHE, with TOF_CO_ varying from 0.34 s^−1^ to 2.1 s^−1^ respectively.

Although the CoPP@CNT composite showed negligible current decay over time, electrodes that were prepared by non-covalent attachment (physically mixed samples) of CoPPCl/CNT–OH with various CoPPCl loadings (CoPPCl/CNT–OH weight ratios of 4.4 × 10^−4^ to 5.6 × 10^−1^) at −0.55 *vs.* RHE showed a 20% decrease in the current density after a 1 hour electrolysis. Not only does covalent grafting improve catalyst stability, but the current density is also enhanced; physically mixed CoPPCl/CNT–OH showed a 50% lower current density at −0.55 *vs.* RHE (1 mA cm^−2^) compared to the covalently grafted CoPP@CNT value (2.1 mA cm^2^). The authors attribute this decrease in current density to catalyst aggregation which is a consequence of non-covalent grafting. The formation of aggregates blocks available active sites on the catalyst and hinder efficient electron transfer, especially at higher catalyst loadings, resulting in lower current densities. Through covalent grafting, the number of immobilized catalysts on the electrode can be optimized, while maintaining a high level of dispersion such that all grafted Co porphyrins are catalytically active.

**Fig. 6:**
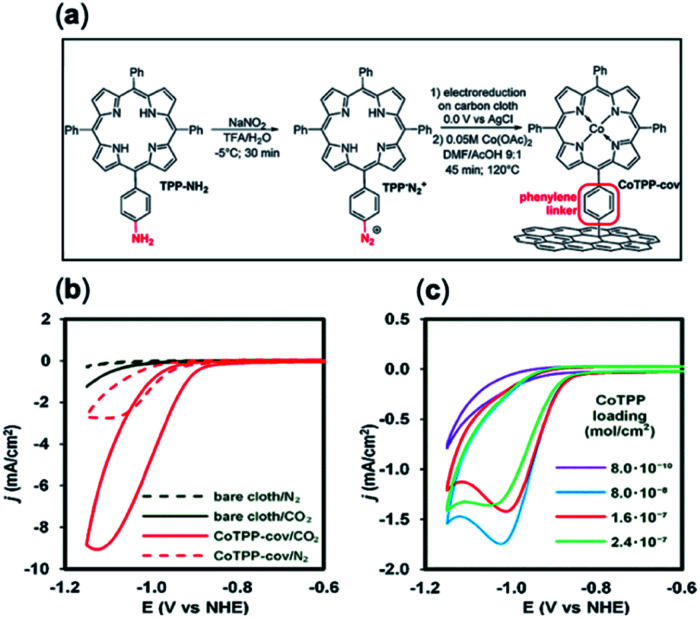
(a) Preparation of covalently immobilized Co tetraphenylporphyrin (CoTPP-cov); (b) cyclic voltammetry (CVs) of CoTPP-cov in N_2_- and CO_2_-purged aqueous solution, CVs of bare carbon cloth are shown for clarity; (c) CV traces of CoTPP-noncov with a variable amount of noncovalently immobilized CoTPP in CO_2_-saturated solution. Conditions: electrolyte: 0.5 M KHCO_3_ in all cases, potential scan rate is 100 mV s^−1^.^105^ Copyright 2019 Elsevier B.V.

Molecules with amine/amide-derived functionalized groups (*e.g.* amines, pyridine linkers) are well positioned for covalent anchoring to a surface through their monodentate axial ligands.^34,104^ Recent approaches have pioneered new techniques whereby a similar effect can be accomplished with organic molecules interfaced with solid supports.^85,133,134^ Marianov *et al.*^105^ have successfully demonstrated direct attachment of porphyrin derivatives (CoTPP-cov) through a aniline-mediated linkage onto glassy carbon (Fig. 6a). In these conditions a higher current density (4.7 mA cm^−2^) was observed compared to their unlinked counterparts (1.4 mA cm^−2^) (Fig. 6b). A positive correlation between the current density and catalyst loading concentration and active surface area was also shown (Fig. 6b and c).

Lessons from homogeneous electrocatalysts have been incorporated into the design of a number of heterogeneous systems. For example, electron donating groups are known to increase the partial negative charge on the metal centre *via* inductive effect resulting in higher CO_2_-to-metal binding energy and enhanced CO_2_RR. Covalent immobilization of an iron tetraphenylporphyrin with six pendant –OH groups in the *ortho* positions and one carboxylic acid group, resulted in a high FE of 92%.^130^ Jiang *et al.*^104^ covalently grafted cobalt tetrakis-(4-aminophenyl)porphyrin (CoTAP) bearing 4 electron-donating amino groups onto a carboxylic acid functionalized CNT *via* an amide linkage. This strategy resulted in an unprecedented ∼100% FE_CO_ at overpotentials of 550 mV and a TOF_CO_ of 6.0 s^−1^, while the non-covalent grafted electrode demonstrated a more moderate FE_CO_ of 85% and TOF_CO_ of 2.3 s^−1^. In comparison to their previous work with covalent and non-covalent grafted cobalt tetraphenylporphyrin (CoTPP), a much lower FE_CO_ was observed in both electrodes; 67% (TOF_CO_ 8.3 s^−1^) for covalent and 52% (TOF_CO_ 4.5 s^−1^) for non-covalent. The authors rationalize this improvement of catalytic activity for several reasons; the presence of electron donating amino groups improves the intrinsic catalytic activity of each individual catalyst, furthermore the amide bond acts as a molecular wire that enhances electron transfer from the CNT to the catalytically active Co centre. Direct covalent connection of the CoTAP to the surface of CNT improves overall reaction rate due to faster electron migration and the diffusion of CO_2_ towards the active centres is no longer hindered by the layers of CoTAP aggregates.

## Electrode immobilization by electropolymerization

Electrode surface immobilization *via* electropolymerization involves a monomer unit consisting of the molecular catalyst and a reactive moiety that undergoes polymerization upon oxidation, which then propagates onto the electrode surface. The oxidation of the monomer can be initiated by chemical means, however electrochemical oxidation grants control of film thickness, the possibility for *in situ* characterization during polymer growth, the lack of complicated purification steps, and most importantly is devoid of toxic oxidants, making this immobilization technique essentially ‘green’.

The polymerization of the film is generally achieved by voltammetrically cycling the monomer in solution at an appropriate potential range and at a controlled sweep rate. Care must be taken to determine the optimal potential for deposition of these films as many have found that they can undergo oxidative degradation at more positive potentials, having negative consequences for the catalytic properties of the film. This technique is demonstrated in one study where the authors use a thiophene ((T)–3,4-ethylenedioxythiophene or EDOT) moiety attached to CoTPP *via* a flexible 1,3-aminothiopropylene spacer, which was electropolymerized into polythiophene on indium-tin-oxide (ITO)-coated glass and carbon paper substrates.^135^ At −0.66 V *vs.* RHE, the Co-porphyrin-based polymer demonstrated a FE_CO_ of 66%, as well as a TOF and TON of 1.6 s^−1^ and 5.7 × 10^3^ respectively, after 1 hour. The polymer film is highly stable and demonstrated a relatively constant current density of 0.936 mA cm^−2^ and FE_CO_ of 36% over the course of a 6 hour controlled potential electrolysis (CPE).

Metal–organic frameworks (MOFs)^136,137^ and covalent organic frameworks (COFs)^138^ introduce more structure and conformation to the aforementioned covalent strategies. Due to the breadth of this field, the topic of (MOFs) and (COFs) is not covered in this review.

## Conclusions and future prospects

As described in this review, electrocatalytic reduction of CO_2_ into fuels and higher value chemicals has become increasingly viable with the advent of recent methodical and technological advances. In order for CO_2_ electroreduction to be industrially viable, electrocatalysts need to perform with both high activity and high selectivity. The use of metalloporphyrins as molecular catalysts has achieved unprecedented results for the reduction of CO_2_ due to their favourable structural and electronic properties. Namely, their structural tuneability enables one to benefit from a wide range of immobilization techniques unavailable to other species. Furthermore, their highly conjugated system allows for enhanced electron conductivity and the ability to tune the electronic structure of the catalytic metal centre. These advantages are further accentuated though immobilization onto heterogeneous electrodes. A number of porphyrin catalysts and their electrocatalytic propensity for CO_2_ electroreduction in heterogeneous systems have been reported herein.

The catalytic activity of these catalysts is strongly dependent on their structural properties and the immobilization technique chosen. Although the goal behind these immobilization techniques is to reduce catalyst aggregation and improve electron transfer from the electrode to the catalyst, the structural complexity of porphyrin molecules coupled with the particular constraints of synthesizing immobilization-compatible molecules hinders rapid development. Advances in structural design allow successful molecules to form stable interactions with the electrode to prevent dissociation, resulting in longer operation capacities.

Despite the variety of optimized heterogeneous molecular catalysts reported so far, there are still limitations which need to be addressed. For commercial electrochemical CO_2_ conversion, it is crucial to achieve a high selectivity of reduction products while ensuring long-term stability of the molecular catalysts. Promising strides in understanding multi-step reaction mechanisms that use molecular catalysts to localize reaction intermediates for reducing CO_2_ to complex C_2_ products is underway.

## Conflicts of interest

There are no conflicts to declare.

## Supplementary Material
